# Recommendations for safer radiotherapy: what’s the message?

**DOI:** 10.3389/fonc.2012.00129

**Published:** 2012-09-28

**Authors:** Peter Dunscombe

**Affiliations:** Department of Oncology, University of Calgary, CalgaryAB, Canada

**Keywords:** radiotherapy, safety, error management

## Abstract

Radiotherapy, with close to a million courses delivered per year in North America, is a very safe and effective intervention for a devastating disease. However, although rare, several deeply regrettable incidents have occurred in radiotherapy and have rightly been the subject of considerable public interest. Partly in response to reports of these incidents a variety of authoritative organizations across the globe has harnessed the expertise amongst their members in attempts to identify the measures that will make radiotherapy safer. While the intentions of all these organizations are clearly good it is challenging for the health care providers in the clinic to know where to start with so much advice coming from so many directions. Through a mapping exercise we have identified commonalities between recommendations made in seven authoritative documents and identified those issues most frequently cited. The documents reviewed contain a total of 117 recommendations. Using the 37 recommendations in “Towards Safer Radiotherapy” as the initial base layer, recommendations in the other documents were mapped, adding to the base layer to accommodate all the recommendations from the additional six documents as necessary. This mapping exercise resulted in the distillation of the original 117 recommendations down to 61 unique recommendations. Twelve topics were identified in three or more of the documents as being pertinent to the improvement of patient safety in radiotherapy. They are, in order of most to least cited: training, staffing, documentation, incident learning, communication, check lists, quality control and preventive maintenance, dosimetric audit, accreditation, minimizing interruptions, prospective risk assessment, and safety culture. This analysis provides guidance for the selection of those activities most likely to enhance safety and quality in radiotherapy based on the frequency of citation in selected recent authoritative literature.

## INTRODUCTION

Over the last several years various august bodies have provided recommendations as to how radiotherapy could be made safer. A consortium of UK professional and other organizations published Towards Safer Radiotherapy (Donaldson, 2007); the World Health Organization published its Radiotherapy Risk Profile (World Health Organization, 2007), and the International Commission on Radiological Protection published Preventing Accidental Exposures from New External Beam Radiation Therapy Technologies (International Commission on Radiological Protection, 2010). In addition there are the established Hierarchy of Actions (a tool for facilitating Health Failure Modes and Effects Analysis) from the US National Centre for Patient Safety (United States Government of Veterans Affairs, 2012), ASTRO’s Target Safely initiative (American Society for Radiation Oncology, 2012a) and, more recently, the Hendee–Herman (H–H) article (Hendee and Herman, 2011) summarizing the recommendations to come out of the AAPM/ASTRO sponsored Miami meeting of 2010. The much anticipated report of the AAPM’s TG 100 (Huq et al., 2008; a comprehensive Failure Modes and Effects Analysis of the Intensity Modulated Radiation Therapy Process) is likely to contain five Key Core Requirements for quality management (of which error management/safety is a significant component). The crude sum of the recommendations emanating from these seven sources is 117.

It is of interest, and of value, to the field of safety in radiotherapy, to look for commonalities between these various recommendations and hence to identify those initiatives that have received the most recognition. Such identification may guide us, as professional organizations, radiotherapy providers, and equipment suppliers, toward those initiatives, considered by the experts in the field, most likely to enhance patient safety in radiotherapy. This communication describes an attempt by the author to distil these 117 recommendations down to a manageable number which have received broad endorsement. Twelve initiatives that are recommended in at least three of the seven documents referred to above have been identified. The means by which these 12 were identified will be described and for each of the twelve a commentary is provided as a basis for further discussion.

## METHODS AND MATERIALS

The seven primary sources of the 117 recommendations listed above constituted the input to the analysis (Donaldson, 2007; World Health Organization, 2007; Huq et al., 2008; International Commission on Radiological Protection, 2010; Hendee and Herman, 2011; American Society for Radiation Oncology, 2012a; United States Government of Veterans Affairs, 2012).

To identify commonalities required mapping each set of recommendations onto a base layer of major topics. There are, of course, several challenges in doing this. Many of the documents referred to above combine two or more recommendations into one numbered paragraph. An example of this is Recommendation 2 in the H–H article (Hendee and Herman, 2011) which includes both the statement “Workstations should be clutter-free...” and “Therapists should not be interrupted while treatments are underway.” While both these recommendations are eminently sensible, the means by which they will be achieved are different. There are many such examples throughout the other documents, mainly but not exclusively, where prose is used to elaborate on the recommendations. On the other hand, however, brevity in the recommendations can lead to difficulty of interpretation. For example, why does “Staff competency assessment” appear as a recommendation in the WHO document (World Health Organization, 2007)? Surely it is a component of “Competency certification” which is recommended in the same document. Maybe two unrelated measures are being recommended here but it is not clear what they are.

These difficulties notwithstanding, the first step was to develop a base layer of topics relevant to patient safety. The starting point for the base layer was the 37 recommendations published in the UK document (Donaldson, 2007) as this was the largest set of recommendations. By topics is meant one to five words which describe the general issue addressed by the recommendation. For example, the UK’s Recommendation 2 led to a base layer topic of “Staffing” and their Recommendation 3 to “Training.” In establishing the initial base layer the problem immediately encountered was that described above of two recommendations being included in one numbered paragraph. Such a problem was identified three times in the UK document (Donaldson, 2007). As an example, Recommendation 5 included checking and interruptions so these were separated. Thus the initial base layer expanded from the original published 37 topics to 40 topics. The next largest group of recommendations (20) is in the H–H article (Hendee and Herman, 2011). These were then mapped onto the initial base layer of 40 topics as far as possible. Eleven of the 20 H–H recommendations could not reasonably be mapped onto the initial base layer derived from the UK recommendations (Donaldson, 2007). Thus, to accommodate these, the base layer had to be expanded to 51 (40 + 11) topics. The inability to map onto the UK document was, in this case, due, in part, to several H–H recommendations being directed principally at vendors about which the UK document had nothing to say. A similar process was followed for the remaining five documents (World Health Organization, 2007; Huq et al., 2008; International Commission on Radiological Protection, 2010; American Society for Radiation Oncology, 2012a; United States Government of Veterans Affairs, 2012). The resulting base layer comprised 61 topics. Put another way, eliminating duplication between the recommendations from the seven sources resulted in 61 unique recommendations.

The “logic” involved in performing this mapping can be illustrated with the example of Training, which was an initial component of the base layer. Recommendation 2 of the UK document (Donaldson, 2007) states that “Training records should be created and maintained...and specific to particular procedures” and “Funding to support training should be available.” ICRP 112 (International Commission on Radiological Protection, 2010), in their second and third recommendations, refer to “a consistent effort on education and training” and “Resources should be allocated so as to avoid substituting proper training...” respectively. These two ICRP recommendations also addressed training but from a different angle than the UK’s Recommendation 2. The other statements that were mapped on to “Training” in the base layer were “Competency certification” and “Staff competency assessment” (World Health Organization, 2007); “Training/additional study/analysis” (United States Government of Veterans Affairs, 2012); “free SAM module” and “educational content into ASTRO meetings” (American Society for Radiation Oncology, 2012a); “Patient safety should be a competency” (Hendee and Herman, 2011), and “Adequate training of staff” (Huq et al., 2008). Thus, for this example, the conclusion is that all seven of the seven sets of recommendations identify “Training,” interpreted as encompassing education, as a general area which is particularly relevant to patient safety.

## RESULTS

The 12 initiatives cited in three or more of these sets of recommendations are given in **Table 1**.

**Table 1:** Citations for the top twelve recommendations.

Recommendation	TSF	WHO	ICRP	NPSF	ASTRO	H–H	TG100
Training	√	√	√	√	√	√	√
Staffing	√	√	√	√		√	√
Documentation/SOP	√		√	√		√	√
Incident learning	√	√	√		√	√	
Communication/	√		√	√		√	
questioning							
Check lists	√	√		√		√	
QC/PM	√	√	√					√
Dosimetric audit	√	√	√			√	
Accreditation	√	√			√	√	
Minimizing	√		√			√	
interruptions							
Prospective risk	√		√			√	
assessment							
Safety culture			√	√		√	

*TSF, Donaldson, 2007; WHO,World Health Organization, 2007; ICRP, International Commission on Radiological Protection, 2010; NPSF, United States Government of Veterans Affairs, 2012; ASTRO, American Society for Radiation Oncology, 2012a; H–H, Hendee and Herman, 2011; TG100, Huq et al., 2008.*

The twelve top recommendations are also listed here with the number of citations in parentheses and the references to the source documents in which the topic appears. In the next section a brief commentary on each of the 12 initiatives is provided as a basis for further discussion within the radiation treatment community.

***Training* (7)** (Donaldson, 2007; World Health Organization, 2007; Huq et al., 2008; International Commission on Radiological Protection, 2010; Hendee and Herman, 2011; American Society for Radiation Oncology, 2012a; United States Government of Veterans Affairs, 2012).

***Staffing/skills mix* (6)** (Donaldson, 2007; World Health Organization, 2007; Huq et al., 2008; International Commission on Radiological Protection, 2010; Hendee and Herman, 2011; United States Government of Veterans Affairs, 2012).

***Documentation/standard operating procedures* (5)** (Donaldson, 2007; Huq et al., 2008; International Commission on Radiological Protection, 2010; Hendee and Herman, 2011; United States Government of Veterans Affairs, 2012).

***Voluntary incident learning system* (5)** (Donaldson, 2007; World Health Organization, 2007; International Commission on Radiological Protection, 2010; Hendee and Herman, 2011; American Society for Radiation Oncology, 2012a).

***Communication/questioning* (4)** (Donaldson, 2007; Huq et al., 2008; International Commission on Radiological Protection, 2010; Hendee and Herman, 2011).

***Check lists – planners and prescribers*(4)** (Donaldson, 2007; World Health Organization, 2007; Hendee and Herman, 2011; United States Government of Veterans Affairs, 2012).

***Quality control and preventive maintenance* (4)** (Donaldson, 2007; World Health Organization, 2007; Huq et al., 2008; International Commission on Radiological Protection, 2010).

***Dosimetric audit* (4)** (Donaldson, 2007; World Health Organization, 2007; International Commission on Radiological Protection, 2010; Hendee and Herman, 2011).

***Accreditation* (4)** (Donaldson, 2007; World Health Organization, 2007; Hendee and Herman, 2011; American Society for Radiation Oncology, 2012a).

***Minimizing interruptions* (3)** (Donaldson, 2007; Hendee and Herman, 2011; United States Government of Veterans Affairs, 2012).

***Prospective risk assessment* (3)** (Donaldson, 2007; International Commission on Radiological Protection, 2010; Hendee and Herman, 2011).

***Safety culture* (3)** (International Commission on Radiological Protection, 2010; Hendee and Herman, 2011; United States Government of Veterans Affairs, 2012).

## COMMENTARY

### TRAINING

Training, interpreted as including education for the purposes of this study, is a recommended initiative in all seven of the sources. However, it is not always clear what the training is in. The UK document does recommend training in Quality Management (Recommendation 33; Donaldson, 2007) but what exactly does this mean? If we desire training to extend beyond education to the development of a specific set of competencies which are thoroughly assessed and a certificate issued then maybe the professional organizations could play a more active and leading role. On-line modules are helpful in this regard (Williamson et al., 2011) but, ideally, they will be supplemented with hands-on activities. Events that incorporate an active component do take place to a limited extent now such as contouring practica and institution run practical workshops on particular technologies and clinical processes. It is, of course, acknowledged that the vendors generally have well developed training programs run by experienced instructors. However, these are obviously geared to the use of the specific equipment which the particular vendor supplies. Perhaps what is required to complement these events is more training in specifically safety related topics, such as human factors, and in process flow, and related failure modes, as they apply to particular processes in a particular clinic. A multidisciplinary approach to such training might mitigate some of the communication difficulties encountered in a busy clinic environment. With practical help from the professional organizations the structure and contents of multidisciplinary specialty certification/credentialing programs could be developed (Brown et al., 2011). It is noteworthy that Training is second from the bottom of the Hierarchy of Actions (United States Government of Veterans Affairs, 2012).

### STAFFING

The only set of recommendations not to mention staffing is that from ASTRO, their Target Safely initiative (American Society for Radiation Oncology, 2012a). However, ASTRO has recently produced Safety Is No Accident (American Society for Radiation Oncology, 2012b) in which staffing is specifically addressed as a significant issue in the context of patient safety. An initiative in this direction is thus actually supported by all seven sources included in this analysis. Whether or not adequate staffing levels are achieved will depend on the willingness of radiotherapy providers to allocate the necessary resources. They are much more likely to do so now that a body such as ASTRO has provided them with up to date staffing recommendations and emphasized the relationship between staffing and patient safety.

### DOCUMENTATION/STANDARD OPERATING PROCEDURES

It is recognized that issues related to documentation/standard operating procedures, their absence or inadequacy, are associated with errors (Clark et al., 2012). Professional bodies, such as the AAPM, have provided the community with a wealth of information and data upon which to base clinic documentation. So why do documentation related errors persist? One reason may be that, while source documents related to the performance expectations of the clinic’s infrastructure have been generated by the professional organizations, documentation of clinical processes, which have to be developed largely locally, is lacking. A second possible reason is that the development of adequate, comprehensive documentation requires the diversion of resources from other activities and, perhaps, this activity has failed to rise to near the top of the priority list of radiotherapy providers. However, it is a common observation that even when adequate documentation does exist it is not always followed. It is unlikely that failure to follow established procedures is for some malicious reason. It is more likely to be due to the procedure either having been forgotten or the significance of not following it not being fully appreciated. Perhaps an FMEA type approach to ranking procedures according to the potential consequences of non-adherence might be of help. For clinical procedures there might be an opportunity for professional organizations and equipment suppliers to work together to identify those that are particularly safety critical. TG 100 is aiming to make a contribution in this area (Huq et al., 2008). However, at the end of the day it is really up to individual institutions to ensure that they have adequate documentation of what they do and how they do it.

### INCIDENT LEARNING

The recent heightened interest in safety in radiotherapy has brought to the fore recognition of the potential benefits of tracking, analyzing, and sharing information on incidents. ROSIS was a pioneer in this area (Cunningham et al., 2010). One of the original architects of ROSIS recently moved on to the International Atomic Energy Agency where he is currently leading the development of an advanced multi-institution incident learning system, SAFRON (International Atomic Energy Agency, 2012). Many institutions are, in parallel, developing their own systems and it will be necessary for all these systems to be able to share their databases in some way if the community is to maximize learning opportunities. The AAPM’s Working Group on the Prevention of Errors has an active subgroup working which, at the time of writing, is concluding a project on defining the generic structure of an incident learning system (Ford, 2012, personal communication). A useful discussion of incident learning systems has been provided by Mutic and Brame (2010).

### COMMUNICATION/QUESTIONING

This topic found its way on to the base layer from the first recommendation in the UK document (Donaldson, 2007). Mapped on to this topic were Time Outs (Hendee and Herman, 2011), “unambiguous, well structured communication” from the ICRP (International Commission on Radiological Protection, 2010), and “clear lines of communication among staff” from TG 100 (Huq, 2012, personal communication). Effective, open communication and respectful questioning could both be regarded as key elements of a well developed safety culture. Whilst professional organizations can certainly emphasize the importance of these behaviors it is up to the leaders of radiotherapy clinics to establish the environment in which communication and questioning are encouraged. An interesting discussion, relevant particularly to the questioning component of this topic, has been given by Low et al. (2010).

### CHECK LISTS

In guarding against the propagation of errors resulting from interruptions, slips, and lapses, check lists clearly have a role. A well known challenge in the use of check lists is automaticity where the checker essentially does a “copy and paste” from the last ten check lists he or she completed. Automating the checking procedure is one way to at least partially overcome this difficulty through the use of commercially available MU calculators or more institution specific developments (Breen and Zhang, 2010). Inevitably there will be a residual amount of hand checking that will have to be done and, whilst not eliminating automaticity, emphasizing those checks which are safety critical may mitigate its effects. The fruits of TG 100’s labors (Huq et al., 2008), together with input from the equipment suppliers, may help to highlight the most important checks.

### QUALITY CONTROL AND PREVENTIVE MAINTENANCE

The AAPM and other professional organizations have provided a plethora of detailed recommendations particularly on the QC of equipment. An opportunity to do more is the QC of processes. TG 100 (Huq et al., 2008) is addressing this aspect of QC although individual facilities will have to customize process QC to their particular operational model.

### DOSIMETRIC AUDIT

There can be little doubt that organizations like the RPC and Equal-ESTRO have had, and continue to have, a huge positive influence on the safety and quality of radiotherapy. Recent incidents have, however, suggested that dosimetric audits are not always carried out appropriately. The first dosimetric audit should take place prior to the first clinical use of the device – obvious but not always followed. And testing the device under conditions other than those used to calibrate it could bring to light errors in subsequent arithmetic manipulations (Cancercare Ontario, 2012).

### ACCREDITATION

Radiation Oncology specific accreditation has so far failed to gain much support in North America (Tripuraneni et al., 2011). Using web based technology the documentation review component of accreditation could be made easier which may entice more institutions to participate. Failing that, stronger “inducements,” such as regulations and tying re-imbursement to accreditation status, may be necessary. Accreditation has proved its value in mammography screening programs in re-assuring the client population of the quality of the service provided. Why would we not adopt a similar approach to restore confidence in our ability to deliver high quality and safe radiotherapy, particularly in view of the press coverage we have received in recent years?

### MINIMIZING INTERRUPTIONS

The issue of interruptions is familiar to all working in a busy clinic. However, it is not always obvious how potentially dangerous interruptions can be when we are in the middle of a critical task (Anthony et al., 2010). Check lists and no interruption zones (Anthony et al., 2010) can be effective strategies in minimizing the risks accompanying interruptions. Awareness of the risks can be enhanced through educational activities organized by professional bodies but it is more likely to be the local, institution led safety culture that will determine whether or not staff members remain respectful of other people’s space.

### PROSPECTIVE RISK ASSESSMENT

This is the approach adopted by TG 100 (Huq et al., 2008) to the rationalization of quality management activities in an environment of stable or diminishing resources and increasingly complex workload. It clearly makes sense to take pre-emptive actions to safeguard safety before introducing new technologies and processes. However, doing so can require knowledge and skills that, in most cases, were not developed during formal training. Professional organizations, such as the AAPM, are doing their bit to promote prospective safety measures (Huq et al., 2008) and their efforts are reinforced by organizations such as the ICRP (International Commission on Radiological Protection, 2010). However, yet again, whether or not prospective risk assessment becomes part of our regular practice will depend upon the commitment of employers and continued leadership within the community.

### SAFETY CULTURE

Many of the topics included in the base layer used for mapping could have been mapped onto this topic but that would not have been helpful for the present analysis. Professional organizations have been explicitly or implicitly supporting the development of a safety culture for many years. However, changing organizational culture is one of the most difficult things to do. What is required is more than involvement from the top: true commitment is what is required. In the context of patient safety, commitment will be evidenced by allocating adequate resources to safety issues which means going beyond the usual platitudes to which we have grown immune.

## DISCUSSION

Clearly “logic” only goes so far in performing this mapping exercise – a significant and variable element of judgment was also employed depending on the recommendations being mapped. The subjectivity in this communication is further evidenced in the commentaries on each of the twelve top ranked initiatives which are clearly based on the author’s opinions.

A second point related to the analysis is that, using the same flexibility in interpretation employed during the main mapping process, some of the 37 recommendations (Donaldson, 2007) used as the base layer topics could have been mapped onto each other. For example, Recommendation 18, which addresses action levels for *in vivo* dosimetry, would have been mapped onto Recommendation 17, which addresses protocols for *in vivo* dosimetry, had the former recommendation come from a different source. For consistency it was decided not to map within the initial base layer established from the UK document (Donaldson, 2007).

A question related to this analysis and the recommendations upon which it based is whether or not there is any evidence supporting the safety initiatives in any of the seven source documents and consequently whether or not they should be weighted unequally. The only source document that comes close to basing its recommendations on evidence is WHO’s Radiotherapy Risk Profile (World Health Organization, 2007). The authors of that document have reviewed reports in the peer reviewed and gray literature of actual events and near misses, performed, as best they could, a Root Cause Analysis and deduced measures that, had they been in place, would have reduced the probability of the event’s occurring. The recommendations in the other six documents represent the consensus opinions of expert groups. There was no justification for weighting the recommendations in these various documents other than equally.

The choice of which recommendations to include in this review was the author’s. No systematic search of the literature was conducted. However, it is believed that all major recent documents with international influence have been included.

Whether it is implementing an incident learning system or enhancing the safety culture within an organization, resources are required. These may be new resources or resources diverted from some other activity thus incurring an opportunity cost. However, as well as debits on the quality/safety balance sheet there will likely also be credits due to a more streamlined, efficient operation and improved staff morale amongst other benefits. Although probably impossible to quantify, the benefits of allocating additional resources to safety/quality issues could well outweigh the costs.

## CONCLUSION

The degree of success of any venture is determined by the quality of its leadership and the resources committed to that venture. The venture for us, at this time, is making radiotherapy safer. As pointed out above, leadership is apparent in several of the areas to which the various recommendations converge with some commitment of resources particularly by the professional organizations. We can do better but we need to focus on those endeavors most likely to be successful. This analysis has attempted to distill the recommendations of many experts down to those initiatives most likely to lead to our desired result – safer radiotherapy.

## Conflict of Interest Statement

The author declares that the research was conducted in the absence of any commercial or financial relationships that could be construed as a potential conflict of interest.
